# Use of machine learning and voice for multiclass classification of Parkinson’s disease, chronic obstructive pulmonary disease, and healthy controls

**DOI:** 10.1038/s41598-026-53409-3

**Published:** 2026-05-19

**Authors:** Alper Idrisoglu, Anders Behrens

**Affiliations:** Blekinge Institute of Technology, Valhallavägen 1, Karlskrona, 371 41 Sweden

**Keywords:** Parkinson’s disease, Chronic obstructive pulmonary disease, Voice analysis, Machine learning, Digital biomarkers, Multiclass classification, Explainable artificial intelligence, Biomarkers, Computational biology and bioinformatics, Diseases, Medical research, Neurology, Neuroscience

## Abstract

Parkinson’s disease (PD) and chronic obstructive pulmonary disease (COPD) are prevalent conditions with substantial impact on quality of life and health care systems. Both disorders affect voice production through different physiological mechanisms, yet neither condition has a widely adopted objective biomarker for routine clinical use. Voice analysis has emerged as a non-invasive digital biomarker candidate, but existing studies have largely focused on binary classification within a single disorder or language. This study aimed to evaluate whether an unified multiclass machine learning (ML) framework applied to sustained vowel “a” phonation can discriminate between PD, COPD, and healthy controls (HC) across linguistically distinct cohorts. Sustained vowel recordings were analyzed from Swedish speaking individuals with COPD and HC, and English-speaking individuals with PD and HC, collected under comparable mobile recording conditions. Acoustic features included baseline voice measures and Mel Frequency Cepstral Coefficients. A soft voting ML framework integrating support vector machine, random forest, CatBoost, and light gradient boosting classifiers was trained using nested cross validation with hyperparameter optimization. Data were partitioned at the participant level into a development cohort and an independent test cohort. Model performance was evaluated using accuracy, macro averaged precision, recall, F1 score, receiver operating characteristic analysis, and confusion matrices. Model interpretability was assessed using Shapley additive explanations and vowel space analysis. The final soft voting classifier achieved robust multiclass discrimination on the participant disjoint independent test set, with an overall accuracy of 0.842 and a macro averaged F1 score of 0.839. Classification performance differed across groups, with the highest performance observed for PD, intermediate performance for HC, and lower performance for COPD. Misclassifications occurred primarily between HC and COPD, while confusion between PD and COPD was minimal. Feature attribution analysis revealed class dependent relevance patterns, and vowel space analysis demonstrated subtle but consistent group level differences. These findings demonstrate the feasibility of using an explainable soft voting machine learning framework applied to sustained vowel phonation to distinguish between neurologically and respiratory driven voice impairments across linguistic contexts. The study supports voice as a promising digital biomarker modality for multiclass clinical discrimination using mobile recordings.

## Introduction

 Parkinson’s Disease (PD) and Chronic Obstructive Pulmonary Disease (COPD) are two prevalent disorders that significantly impact patients’ quality of life^1,2^. Parkinson’s Disease, a neurodegenerative disorder, primarily affects motor function, causing tremors, rigidity, and bradykinesia, along with a range of non-motor symptoms^3^. On the other hand, COPD, a progressive lung disease characterized by airflow obstruction, leads to breathing difficulties and persistent respiratory symptoms^4,5^. COPD and PD are widespread and burdensome conditions with high societal costs^1,2^. PD is the fastest-growing neurological disorder worldwide^3,6^, while COPD is among the leading causes of chronic morbidity and mortality^4,5^. Both PD and COPD have an insidious onset, with a prolonged prodromal phase before the development of overt symptoms. The diagnosis in both conditions primarily relies on clinical assessment, which makes accurate identification particularly difficult in the early stages of disease, making the development of sensitive objective biomarkers an urgent need. Beyond these dominant clinical manifestations, namely motor impairment in PD and respiratory impairment in COPD, Parkinson’s disease is frequently associated with profound voice and speech disorders in up to 90% of individuals as the disease progresses^7^. The COPD manifestations also extend to vocal behavior due to obstructed airflow from the lungs^8^.

As aforementioned, both PD and COPD also profoundly affect the vocal apparatus. In PD, neurological degeneration disrupts the motor control of speech muscles, resulting in hypophonia, monotonic speech, and reduced speech clarity^9–11^. Meanwhile, in COPD, the diminished lung function and airway obstruction alter the airflow required for speech production, often leading to breathy, hoarse, or weak voices^12–14^. These changes can result in overlapping acoustic patterns, such as reduced loudness and irregular phonation, while disease-specific markers may also exist. Distinguishing between shared and unique features is therefore relevant not primarily because COPD and PD represent a common direct differential-diagnostic pair, but because it allows evaluation of whether voice-based biomarkers capture disease-informative acoustic patterns rather than merely separating healthy from abnormal voice, while also providing an initial basis for assessing whether such patterns may generalize across different linguistic contexts.

Given these impacts, voice analysis has emerged as a non-invasive method for assessing and potentially diagnosing these conditions. In particular, the analysis of sustained vowel utterances, such as phonation of the vowel “a,” provides valuable insights into vocal quality^15,16^ and offers a comparable speech task through which voice deviations associated with both conditions can be examined within a common analytical framework. This study leverages baseline acoustic features (such as pitch, jitter, and shimmer) and Mel-Frequency Cepstral Coefficients (MFCCs), which are widely used in speech and audio processing, to capture the detailed spectral properties of voice signals^17^.

In addition to the developments in using voice as a biomarker, recent advancements in machine learning (ML) have revolutionized the field of medical diagnostics, enabling the analysis of complex and high-dimensional data to uncover patterns that may not be apparent through traditional methods^18^. ML techniques, particularly those involving ensemble of multiple classifiers into one, have shown great promise in improving the accuracy and reliability of diagnostic tools^19,20^ However, many studies report the superiority of different single ML models when applied to vocal biomarker detection for neurological disorders, such as Parkinson’s disease, and respiratory conditions, like COPD^21–25^. This disparity in reported performance often arises from variations in data acquisition, feature extraction methodologies, and the specific machine learning architectures employed^26^. In addition to that, the majority of experiments invoke binary classification between healthy controls and a particular disease and may therefore demonstrate the ability to differentiate normal from abnormal voice^21^. It is less clear whether ML methods can distinguish between different conditions affecting voice through different physiological mechanisms, particularly when the data also originate from different linguistic contexts.

Building on this background, the present study extends the exploration of voice as a digital biomarker by integrating data from linguistically and clinically distinct cohorts. The COPD dataset includes participants who speak Swedish, while the mPower dataset comprises individuals with PD who speak English. This multi-linguistic and cross-condition design enables the examination of both shared and disease-specific acoustic characteristics across disorders that affect phonation through different physiological mechanisms. Although the mechanisms discussed earlier differ, both conditions disrupt the integrated control of respiration, phonation, and articulation, leading to overlapping acoustic signatures such as reduced vocal intensity and irregular phonatory patterns mentioned earlier in the text.

These similarities highlight the need to consider PD and COPD within a broader conceptual framework of *voice-affecting disorders*. This perspective aligns with the *Classification Manual for Voice Disorders–I*^27^, which organizes voice disorders based on physiological origin rather than diagnostic labels. Within this framework, voice disorders are grouped into neurogenic, structural, and functional categories, with PD and COPD representing distinct yet intersecting neurogenic and respiratory subdomains affecting phonatory and aerodynamic control. Subsequent reviews have emphasized that adopting such mechanistic classifications enhances diagnostic precision and facilitates cross-condition comparisons^14^.

To address these complexities, the current work presents a *proof-of-concept study* that applies an ensemble learning approach based on a soft-voting classifier, integrating Support Vector Machine (SVM), Random Forest (RF), CatBoost (CB), and Light Gradient Boosting (LGB) ML classifiers. This strategy combines complementary model properties to improve classification performance and generalization across heterogeneous datasets. The objective is to assess whether an ensemble-based framework can effectively discriminate between PD, COPD, and healthy controls (HC) using Base Line Acoustic (BLA) features such as pitch, jitter, shimmer, and MFCCs.

By uniting data from distinct linguistic contexts and physiological domains, the study contributes to a broader understanding of voice-affecting disorders as a multidimensional spectrum rather than isolated disease entities. Beyond classification performance, the study incorporates SHapley Additive exPlanations (SHAP) to interpret feature contributions and enhance model transparency. This approach allows the identification of the most influential acoustic parameters driving model predictions, thereby linking algorithmic outcomes with physiologically interpretable voice characteristics. The integration of SHAP provides insight into both shared and condition-specific vocal patterns and strengthens the interpretability of the proposed ensemble model. The findings are expected to contribute to the identification of robust and generalizable acoustic markers and to support the development of objective, explainable, voice-based diagnostic tools across neurological and respiratory conditions.

## Related work

Studies investigating the use of voice as a biomarker for assessing PD and COPD are increasing in the recent literature^11,21,28^. Recent reviews have summarized progress in applying voice analysis and ML for assessing disorders that influence phonation. A comprehensive systematic literature review examined studies applying ML to voice-based diagnostics and monitoring^21^. The review revealed that most research focused on PD, while respiratory diseases, such as COPD, were rarely represented. The authors highlighted variability in feature extraction, limited dataset standardization, and the absence of studies comparing neurologically and respiratory-driven voice disorders within the same analytical framework.

Few studies have investigated multiclass voice-based classification across clinical conditions^21,29–32^. Most existing research is limited to binary frameworks, typically comparing patients with HC^26,33^. Within the neurological domain, multiclass analyses have been used to distinguish between different dysarthric conditions, for instance, between hypokinetic and ataxic dysarthria, demonstrating that acoustic features can separate neurogenic voice impairments based on their underlying physiological mechanisms^11,34^. However, no published study has to date examined multiclass classification involving PD, COPD, and HC despite these conditions producing overlapping alterations in phonation such as reduced intensity, irregular phonation, and instability of fundamental frequency^35,36^.

Multiclass approaches, such as assembling several ML classifiers for binary classification and training models to distinguish several conditions regardless of languages, offer the potential to address this limitation by revealing both shared and disorder-specific acoustic patterns^37,38^. Such models have been explored for differentiating among several dysarthria types or between PD and other neurogenic speech disorders^39^, yet they have not been extended to include respiratory disorders like COPD. Consequently, there is a lack of evidence on how neurological and respiratory mechanisms jointly influence vocal acoustics within a unified analytical framework.

The present study aims to address this gap by integrating voice data from the Swedish COPDVD dataset^40^, representing individuals with COPD and healthy controls, and the English mPower dataset^41^, containing PD and control recordings. Both datasets were collected through mobile devices under naturalistic conditions, providing comparable data sources. By applying a multiclass ensemble-learning framework and explainability analysis through SHAP, the study evaluates whether shared and condition-specific vocal features can be distinguished across neurological and respiratory domains.

## Materials and methods

**Fig. 1:**
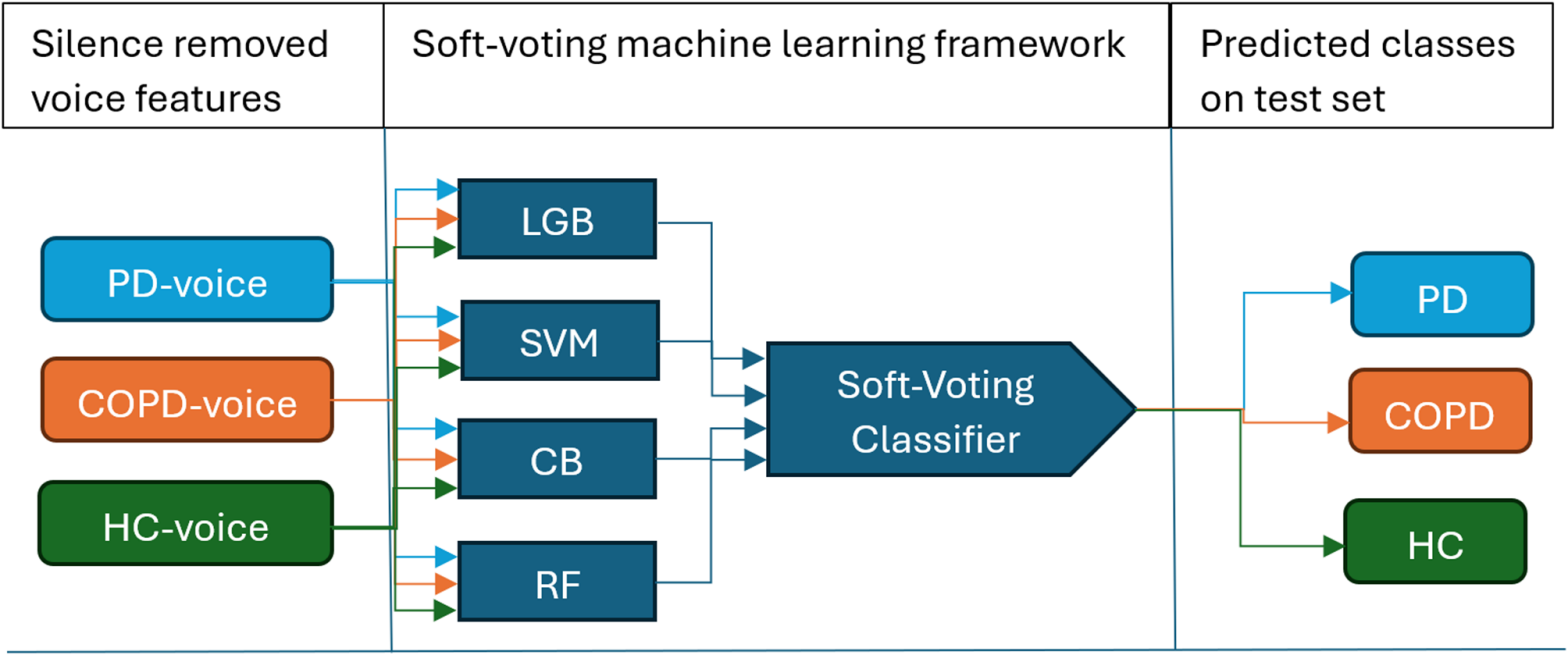
General workflow of the proposed soft-voting machine learning framework for multiclass voice classification.

This section describes the material and methodological procedures used in the present study. Two datasets were utilized due to their similar recording conditions and sustained-vowel “a” tasks. The first dataset originated from the mPower project, released by Sage Bionetworks through the Synapse platform^41^. The second dataset, COPDVD, was collected at Blekinge Institute of Technology (BTH) through the research and education clinic^40^. Figure 1 provides an overview of the study workflow. Silence-removed sustained-vowel recordings were transformed into acoustic feature vectors and evaluated using a soft-voting machine learning framework combining LGB, SVM, CB, and RF. The ensemble aggregated class-probability outputs from the base classifiers to produce final test-set predictions for PD, COPD, and HC.

### mPower dataset

The mPower dataset consists of English-speaking participants who provided self-reported information regarding PD or HC status^41^. Participants recorded a sustained vowel “a” for approximately 10 seconds using a mobile application. The dataset includes 6022 recordings from 5826 individuals, including 968 PD and 3972 HC participants. Although the dataset contains additional voice tasks such as counting and tapping, only sustained vowel “a” recordings and demographic variables were used in this study to ensure comparability with the COPDVD dataset.

### COPDVD dataset

The COPDVD dataset comprises sustained vowel “a” recordings from Swedish-speaking participants recruited at BTH. A total of 1246 recordings were collected from 68 individuals, including 30 with chronic obstructive pulmonary disease (COPD) and 38 HC participants^40^. In contrast to the mPower dataset, the COPD labels in COPDVD were derived from the clinically diagnosed COPD cohort rather than from self-report alone. Although the original COPDVD study included additional clinical, severity-related, and questionnaire-based information, only sustained vowel “a” recordings and demographic information were analyzed in the present study to maintain comparability with the mPower dataset.

### Data harmonization and feature extraction

Because the mPower dataset contained substantially more participants than COPDVD, harmonization procedures were applied to create demographically comparable groups. Participants were matched on age, gender, and number of recordings. From the COPDVD dataset, 24 (12 Female, 12 Male) individuals with COPD and 24 (12 Female, 12 Male) HC participants were selected. From the mPower dataset, 24 (12 Female, 12 Male) PD and 24 (12 Female, 12 Male) HC participants were chosen using identical matching criteria. In total, 96 participants contributed 1723 usable sustained vowel “a” recordings.

The matched groups showed similar age distributions. In COPDVD, the average ages were 72.04 years (standard deviation 6.86) for the COPD group and 72.13 years (standard deviation 6.84) for the HC group. In mPower, the matched PD group averaged 70.40 years (standard deviation 6.32), and the matched HC group averaged 71.39 years (standard deviation 7.22). This procedure ensured demographic comparability and reduced the risk of age-related confounding.

To prevent subject leakage in the final generalization assessment, the matched dataset was partitioned at the participant level into a development cohort and an independent test cohort. After harmonization, the dataset comprised 96 participants in total: 24 COPD participants from COPDVD, 24 PD participants from mPower, and 48 healthy controls, including 24 healthy controls from each dataset. Of these, 72 participants were allocated to the development cohort and 24 to the independent test cohort. No participant contributed recordings to both cohorts. Within the development cohort, recordings were further split at the recording level into training and validation subsets, meaning that some participants contributed recordings to both subsets. Thus, training and validation subsets were not participant-disjoint, whereas the independent test subset was fully participant-disjoint. In total, 72 participants contributed recordings to the development cohort (training/validation), and 24 participants contributed recordings to the independent test cohort. This yielded 1155 recordings for training, 289 recordings for validation, and 279 recordings for independent testing. This corresponds to a 75% versus 25% participant-level split, which lies within the commonly used range in machine learning research, where approximately 70–80% of data are allocated for model development, and 20–30% are reserved for independent testing^42–44^. This ratio strikes a balance between providing sufficient training material for stable model learning and maintaining a robust, independent test group for assessing generalization.

All recordings were trimmed to remove silence at the onset and offset using an energy-based voice activity detection procedure with a moving-average filter, implemented in Python using librosa and soundfile, as described in the original work^40^. The remaining voice-active portion was analyzed to extract baseline acoustic and spectral features. These included jitter, shimmer, fundamental frequency, formant frequencies, and MFCCs with their first and second derivatives. Each recording yielded a 102-dimensional feature vector, which also included the demographic variables listed in Table 3 in the Appendix. The duration of active phonation was excluded to avoid bias attributable to the fixed recording length present in the mPower dataset. These pre-extracted acoustic feature vectors were then used as input to the machine learning models described below.

### Machine learning framework

The machine learning pipeline was designed to ensure unbiased generalization estimates and reproducibility. Models were trained using recordings from the development cohort. Hyperparameter optimization and model selection were performed within the nested cross-validation framework applied to the development cohort. In addition, a recording-level validation subset (with participant overlap with training) was used for secondary performance reporting and diagnostic checks. The independent test subset, which was fully participant-disjoint, was reserved exclusively for the final generalization assessment.

Nested cross-validation was employed to avoid information leakage during hyperparameter optimization. This procedure separates model selection from model evaluation and is recognized as a reliable framework for performance estimation when tuning is required^45,46^. In this study, the outer loop interval varied from two to ten folds, with each outer fold paired with an inner loop of identical structure. Each unique outer–inner configuration constituted an independent experiment. Hyperparameter optimization was carried out only on the training portion of each outer fold, and the optimized models were evaluated on the corresponding held-out partition.

Four supervised ML classifiers were implemented in Python. The SVM and RF models were implemented using scikit-learn, the CB model was implemented using the catboost package, and the LGB model was implemented using the lightgbm package. SVM was included because of its strong theoretical foundations and established performance on high-dimensional biomedical data^47^. RF were selected due to its robustness and ability to reduce variance through the aggregation of multiple decision trees^48^. CB was used because of its ordered boosting strategy and efficient GPU implementation, which are designed to reduce prediction shift and overfitting in tabular data^49–51^. Light Gradient Boosting (LGB) was included because its histogram-based gradient boosting and leaf-wise tree growth provide fast and memory-efficient training with GPU support^52^.

Each classifier was tuned using a predefined hyperparameter gridsearch evaluated within the inner loop. RF hyperparameters included number of trees (n_estimators: 50, 100, 200), maximum tree depth (max_depth: None, 10, 20), and minimum samples required to split an internal node (min_samples_split: 2, 5, 10). Support Vector Machine hyperparameters included the regularisation parameter (C: 0.1, 1, 10), kernel type (linear, radial basis function, polynomial), and polynomial degree (degree: 2, 3, 4). CatBoost hyperparameters included number of boosting iterations (iterations: 100, 200, 300), learning rate (0.01, 0.1, 0.2), tree depth (4, 6, 8), and L2 regularisation (l2_leaf_reg: 1, 3, 5). LGB hyperparameters included number of estimators (n_estimators: 50, 100, 200), learning rate (0.01, 0.1, 0.2), number of leaves (num_leaves: 20, 31, 50), maximum depth (max_depth: −1, 10, 20), L1 regularisation (lambda_l1: 0, 0.1, 0.2), and minimum child samples (min_child_samples: 20, 30, 40). SVM and RF were trained using CPU resources, whereas CB and LGB were trained on GPU to improve computational efficiency. After optimal hyperparameters were identified, each classifier was retrained on the full training portion of the corresponding outer fold.

To increase robustness, the optimized classifiers were combined into a soft-voting ensemble, in which each model contributed class-probability estimates that were averaged to determine the final predicted class. Ensemble learning has been shown to improve classification performance and stability when combining diverse model families^53^. In the present work, the ensemble integrated the tuned SVM, RF, CB and LGB models.

Because the nested cross validation involved repeated training of GPU-based models, a multiprocessing strategy was used to maintain computational feasibility. Each configuration was executed in parallel as an independent worker process. GPUs were assigned sequentially in a round-robin manner and isolated at process level to prevent resource contention.

After all nested configurations were completed, the final ensemble model was evaluated on both the validation subset and the independent test subset. Evaluation metrics included accuracy, macro precision, macro recall, macro F1-score, per-class classification reports and confusion matrices. All trained models, hyperparameter configurations and background samples for subsequent explainability analyses were stored to ensure reproducibility.

### Ethical considerations

All data used in this study were collected in accordance with applicable ethical regulations. The COPDVD dataset was obtained under approval from the Swedish Ethical Review Authority (DNR: 2020 − 01045), and all participants provided written informed consent prior to participation. The mPower dataset was accessed as a publicly available, fully de-identified dataset collected with appropriate ethical oversight and informed consent. The present analyses involved secondary use of de-identified data only.

### Analysis

#### Classification performance evaluation

Model performance was evaluated using metrics appropriate for multiclass clinical classification involving PD, COPD, and HC. Overall classification accuracy was used as a primary summary measure. Because the dataset was longitudinal and participants contributed different numbers of repeated voice recordings over time, residual imbalance remained across the training, validation, and independent test subsets in terms of the number of recordings contributed per participant, despite prior participant-level harmonization. To ensure balanced evaluation across diagnostic groups, macro-averaged precision, recall, and F1-score were computed, assigning equal weight to each class regardless of sample size. Macro-averaging is recommended in biomedical multiclass settings to mitigate the influence of class imbalance and to provide clinically interpretable performance estimates across all diagnostic categories^54^. Performance metrics were computed separately for the training, validation, and independent test subsets obtained through nested cross-validation. This separation enabled assessment of model fitting, hyperparameter stability, and generalization performance.

#### Class-specific error analysis

To characterize class-wise performance and misclassification patterns, confusion matrices were examined for all data subsets. Confusion matrices were reported both as absolute counts and as row-normalized proportions. Row-normalized matrices were used to estimate class-conditional recall and to facilitate clinical interpretation of diagnostic error patterns, particularly misclassifications between HC and COPD, and between PD and HC^55^.

#### Discrimination analysis

To further assess discriminative ability independent of a fixed decision threshold, one-vs-rest receiver operating characteristic (ROC) curves and area under the curve (AUC) were computed for each class, along with macro- and micro-averaged summaries^56^. Because ROC curves can be overly optimistic in imbalanced or clinically asymmetric settings, precision–recall (PR) curves were additionally analyzed to evaluate the trade-off between sensitivity and positive predictive value^57^.

#### Ensemble contribution and robustness analysis

To assess ensemble robustness and the relative contribution of individual base learners, a Leave One Model Out (LOMO) analysis was performed. Ensemble performance was recalculated after removing each base model in turn, and the resulting change in classification accuracy was quantified. This approach provides insight into model redundancy, diversity, and the extent to which specific learners drive ensemble performance^53,58^. In addition, the influence of each base learner on probabilistic predictions was assessed by computing the mean absolute deviation between each model’s predicted class probabilities and the ensemble-averaged probabilities. Larger deviations indicate greater influence on the ensemble’s final output.

#### Model interpretability and feature attribution

To support interpretability, SHAP values were used to quantify feature contributions to model predictions. SHAP values were computed for the final soft-voting classifier and examined separately for HC, COPD, and PD recordings. Feature importance was evaluated based on both magnitude and direction of SHAP values, enabling assessment of how specific acoustic features contribute differently across diagnostic groups^59,60^. Feature attributions were analyzed at the global level and stratified by true class label to examine class-dependent relevance patterns and support physiologically and clinically meaningful interpretations of model behavior.

#### Acoustic–physiological analysis of vowel space

In addition to all aforementioned performance metrics, to provide a physiologically interpretable acoustic perspective, vowel-space analysis based on the first and second formant frequencies (F1–F2) was conducted. Vowel-space geometry is a well-established proxy for articulatory configuration and vocal tract function and has been widely used in clinical phonetics and speech pathology research^61^.

## Results

All results reported in this section correspond to the soft-voting classifier that achieved the best overall performance during the nested cross-validation model selection procedure. Nested cross-validation configurations were evaluated by varying both the outer and inner loop fold numbers from 2 to 10. Among these configurations, the 3-fold outer loop with a 10-fold inner loop yielded the highest overall performance during grid-search hyperparameter optimization and was therefore selected as the final model for all subsequent analyses. The final ensemble combined the optimally tuned SVM, RF, CB, and LGB base classifiers.

### Ensemble configuration and hyperparameter selection

The final classification model consisted of a soft-voting classifier integrating SVM, RF, CB, and LGB. Hyperparameters for each base learner were optimized within the nested cross-validation framework described in the Methods section. Table 1 reports the selected optimal hyperparameters for each base model in the final soft-voting classifier, which together define the ensemble configuration used for all subsequent analyses.

**Table 1:** Optimal hyperparameters for the final ensemble configuration.

Model	Hyperparameter	Selected value
Support Vector Machine	C	0.1
	kernel	linear
	degree	2
Random Forest	n_estimators	50
	max_depth	20
	min_samples_split	2
CatBoost	iterations	200
	learning_rate	0.1
	depth	8
	l2_leaf_reg	1
LGB	n_estimators	100
	learning_rate	0.2
	num_leaves	20
	max_depth	−1
	lambda_l1	0
	min_child_samples	30

### Overall multiclass classification performance

Overall multiclass classification performance is summarized in Table 2. On the independent test subset, the ensemble achieved an overall accuracy of 0.842, with a macro-averaged precision of 0.852, macro-averaged recall of 0.829, and macro-average F1-score of 0.839. Performance on the validation subset was higher, with an accuracy of 0.976 and a macro-average F1-score of 0.975. Training performance reached an accuracy of 0.981 with a macro-average F1-score of 0.980. Class-specific results on the independent test subset showed that Parkinson’s disease achieved the highest F1-score (0.915), followed by healthy controls (0.839) and chronic obstructive pulmonary disease (0.763). Precision for Parkinson’s disease was high (0.959), while healthy controls showed higher recall (0.878) than precision. Chronic obstructive pulmonary disease showed lower recall (0.735) compared with the other classes. Across the training and validation subsets, class-wise precision, recall, and F1-scores were consistently high for all diagnostic groups, with limited variation between classes (Table 3).

**Table 2:** Multiclass classification performance for training, validation, and independent test sets.

Dataset	Class	Precision	Recall	F1-score	Support
Training	HC	0.9826	0.9842	0.9834	633
	COPD	0.9756	0.9689	0.9722	289
	PD	0.9829	0.9871	0.9850	233
	Accuracy			**0.9810**	1155
	Macro avg	0.9804	0.9801	0.9802	1155
Validation	HC	0.9748	0.9810	0.9779	158
	COPD	0.9718	0.9583	0.9650	72
	PD	0.9831	0.9831	0.9831	59
	Accuracy			**0.9758**	289
	Macro avg	0.9766	0.9741	0.9753	289
Test	HC	0.8042	0.8779	0.8394	131
	COPD	0.7937	0.7353	0.7634	68
	PD	0.9589	0.8750	0.9150	80
	Accuracy			**0.8423**	279
	Macro avg	0.8523	0.8294	0.8393	279

### Discrimination performance and confusion matrix results

**Fig. 2:**
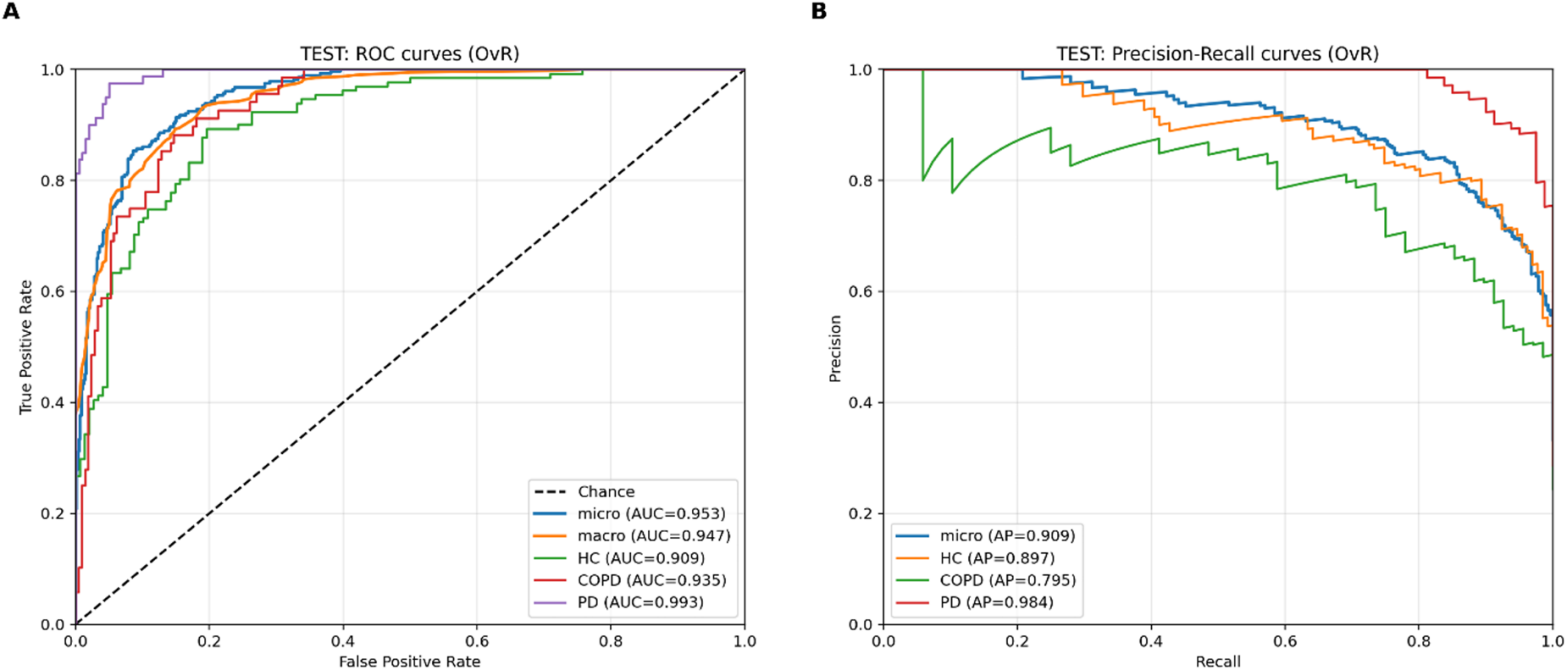
Discrimination performance of the soft-voting classifier model on the independent test set. (**A**) One-vs-rest ROC curves for HC, COPD, and PD with micro- and macro-averaged summaries. (**B**) One-vs-rest precision–recall curves for HC, COPD, and PD with a micro-averaged summary.

Figure 2 shows the discrimination performance of the ensemble model on the independent test set. Panel A presents one-vs-rest ROC curves, with a micro-average AUC of 0.953 and a macro-averaged AUC of 0.947, indicating high overall class separability. Class-specific AUC values were 0.993 for PD, 0.935 for COPD, and 0.909 for HC. Panel B presents one-vs-rest precision–recall curves, where average precision was highest for PD (0.984), followed by HC (0.897) and COPD (0.795). The precision–recall curves show that precision decreases with increasing recall for all classes, with a more pronounced decline for COPD compared with HC and PD.

**Fig. 3:**
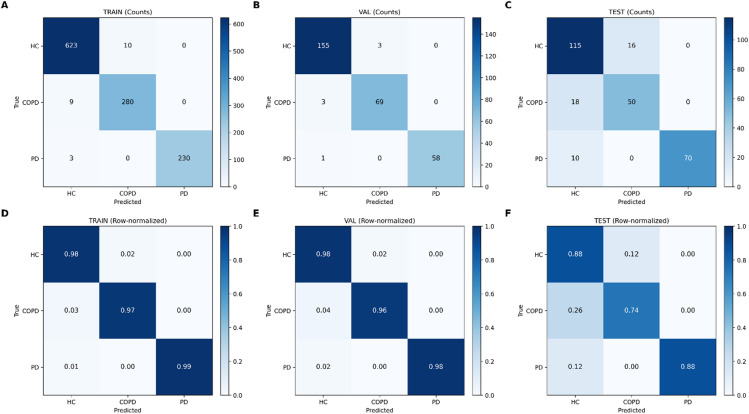
Confusion matrices for the final soft-voting classifier model on the training, validation, and independent test sets. Panels **A**–**C** show absolute classification counts, and Panels **D**–**F** show the corresponding row-normalized matrices. Rows indicate true class labels and columns indicate predicted class labels for HC, COPD, and PD.

Figure 3 presents the confusion matrices for the final ensemble model evaluated on the training, validation, and independent test sets, shown both as absolute counts (Panels A–C) and as row-normalized proportions (Panels D–F). On the training set (Panels A and D), the majority of recordings in each class were correctly classified, with limited off-diagonal entries. HC recordings were predominantly classified as HC, with a small number misclassified as COPD. COPD recordings were largely classified as COPD, with a small number misclassified as HC. PD recordings showed minimal misclassification, with a small number assigned to HC and none assigned to COPD. A similar pattern was observed on the validation set (Panels B and E), where most recordings across all three classes were correctly classified. Misclassifications were primarily observed between HC and COPD, while PD recordings were almost exclusively classified as PD. The row-normalized validation matrix shows high class-specific recall across all classes. On the independent test set (Panels C and F), correct classifications remained dominant for all classes, though increased off-diagonal entries were observed compared with the training and validation sets. Misclassifications were again primarily observed between HC and COPD in both directions. PD recordings were largely classified as PD, with a small proportion misclassified as HC and none misclassified as COPD. The row-normalized test matrix illustrates reduced recall for COPD relative to HC and PD, while PD remained high.

**Fig. 4:**
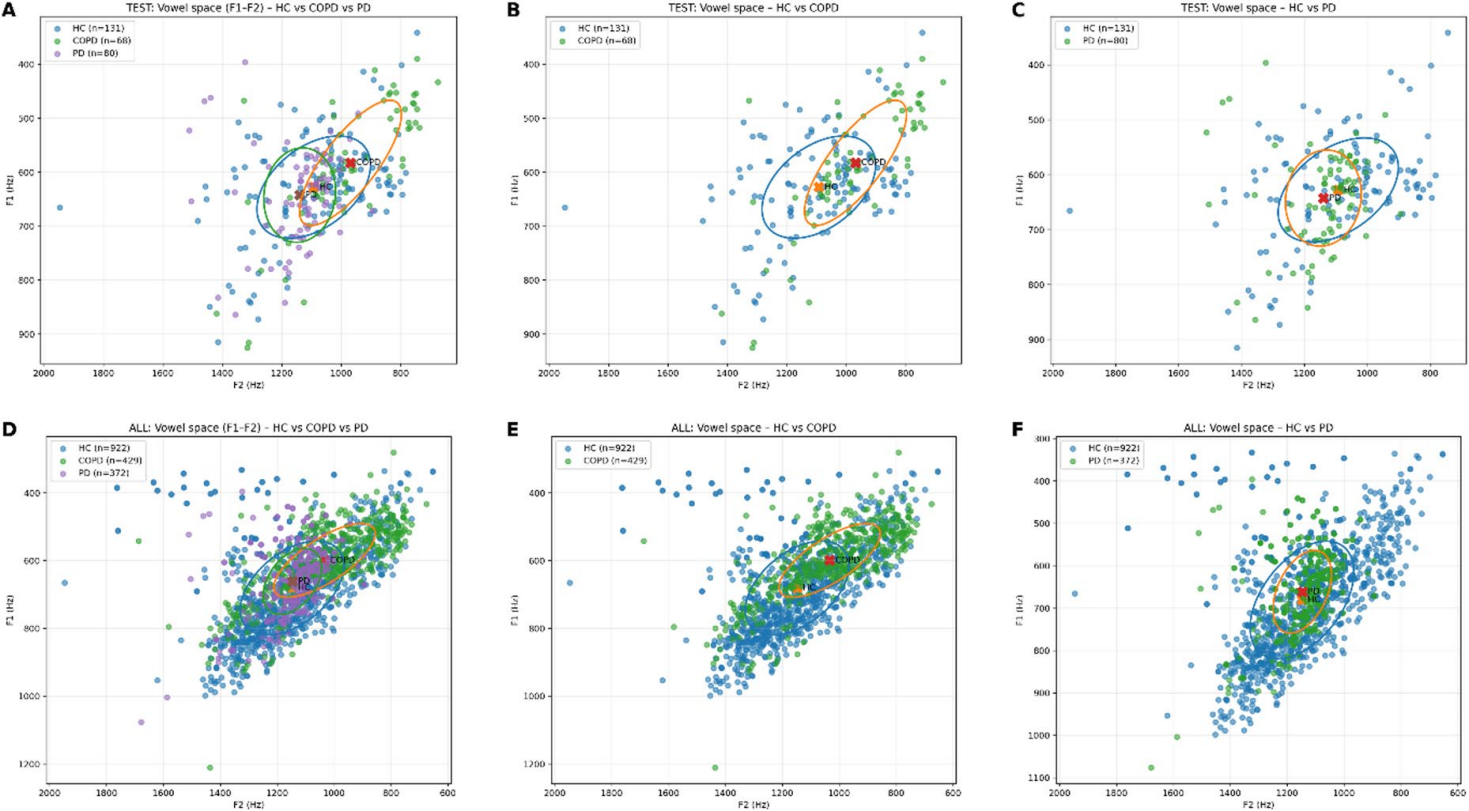
Vowel space distributions (F1–F2) for HC, COPD, and PD in the test set and the combined dataset.

Figure 4 illustrates the distribution of vowel space (F1–F2) across HC, COPD, and PD for the test set (A–C) and the combined dataset (D–F). In the three-class setting (A, D), substantial overlap is observed among the groups; however, systematic differences in centroid location and dispersion are evident. COPD recordings show a displacement toward lower F2 and slightly lower F1 values relative to HC, while PD recordings are positioned closer to HC but exhibit increased variability. In the pairwise comparisons, clearer group-specific patterns emerge. In HC vs. COPD (B, E), the COPD centroid is shifted relative to HC, accompanied by an elongated dispersion reflecting greater within-class variability. In HC vs. PD (C, F), the centroids of HC and PD are closer in the F1–F2 plane, with PD showing a more compact distribution in the test set and increased overlap in the combined dataset. Across all comparisons, the spatial organization observed in the test set closely mirrors that of the combined dataset. Relative centroid positions, orientation of the vowel space distributions, and degrees of overlap are preserved between datasets, indicating that the test set is representative of the overall vowel space structure. Overall, Fig. 4 demonstrates that group-level differences are present but subtle, characterized primarily by shifts in centroid location and differences in dispersion rather than by clear separation in the F1–F2 plane.

### Vowel space characteristics

Because the compared cohorts were drawn from different language backgrounds, part of the observed F1-F2 variation, including the centroid shifts and dispersion differences seen in Fig. 4, may reflect language-specific articulatory characteristics in addition to disease-related effects. However, the use of sustained vowel phonation reduces lexical and prosodic variability and therefore provides a more controlled basis for cross-condition comparison.

**Fig. 5:**
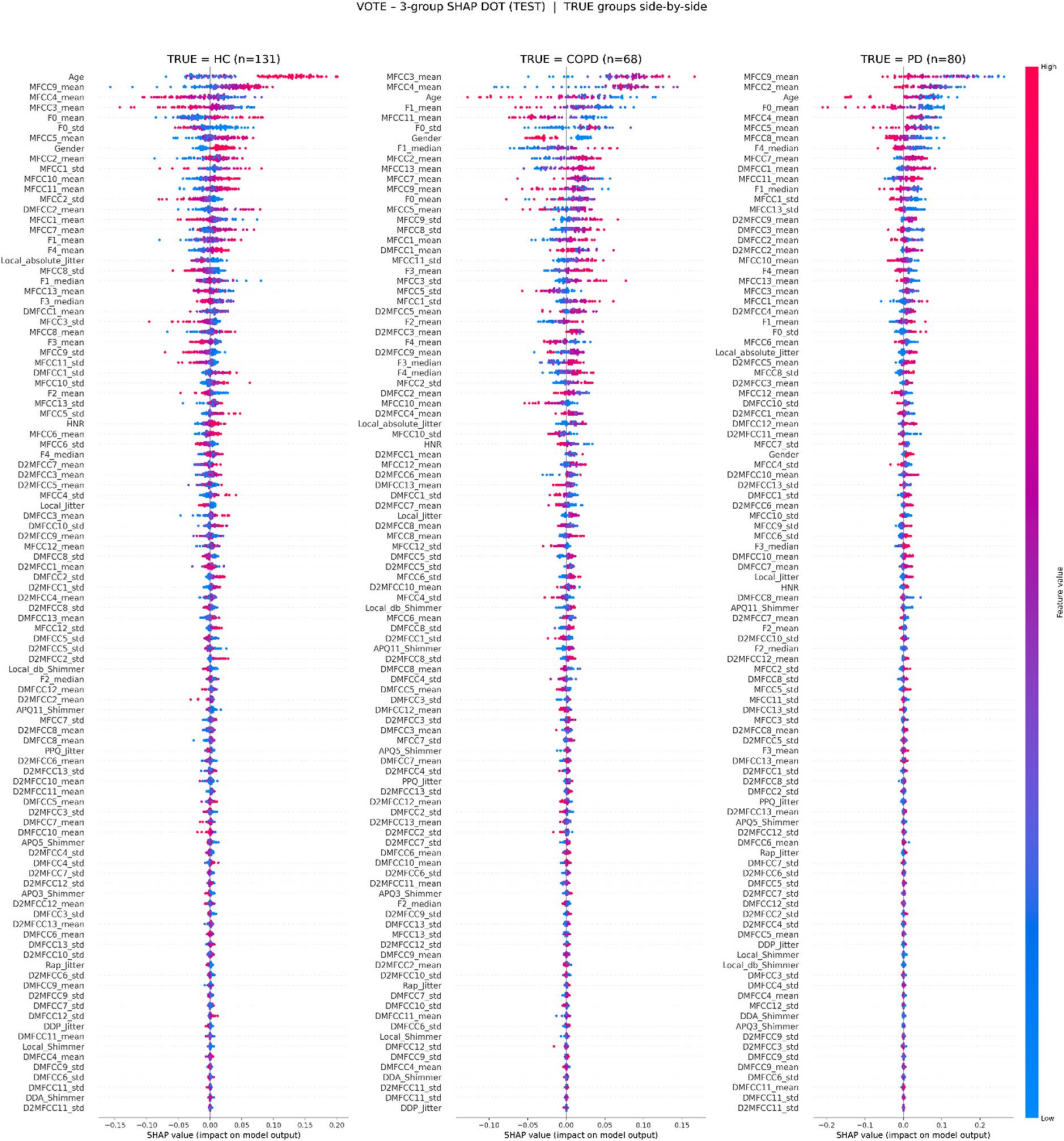
SHAP feature importance representation of different test groups (HC, COPD, and PD) in relation to the soft-voting classification.

Regarding feature importance, Fig. 5 illustrates the order, magnitude, and direction of the 102 features used for testing the soft-voting classifier, as determined by SHAP values. The figure presents class-specific SHAP distributions for HC, COPD, and PD, allowing direct comparison of feature contribution patterns across the three groups. The results show that feature importance rankings and contribution directions differ markedly between groups, indicating that distinct sets of features dominate the model output depending on the true class. In that regard, the SHAP results demonstrate that the soft-voting classifier assigns importance to different subsets of features depending on the diagnostic group. While age, MFCC-based spectral descriptors, and pitch-related measures are consistently present among the influential features, their relative ranking and contribution directions vary across healthy controls, COPD, and Parkinson’s disease.

### SHAP-based feature importance and base learner contribution to ensemble performance

In the HC subgroup (TRUE = HC, *n* = 131), age represents the most influential feature, followed by several spectral envelope descriptors, primarily MFCC-based measures. Pitch-related features, including measures of fundamental frequency level and variability, also appear among the highest-ranked contributors. These features show broad SHAP value distributions, reflecting substantial variability in their impact across individual recordings and speakers in the dataset. Beyond the top-ranked features, the remaining variables display progressively smaller SHAP magnitudes and are largely centered around zero.

For the COPD subgroup (TRUE = COPD, *n* = 68), the highest-ranked features are dominated by MFCC-derived parameters, with MFCC3_mean and MFCC4_mean showing the largest absolute SHAP values. Age remains among the leading contributors, while formant-related measures, particularly first-formant statistics, are consistently ranked higher than in the healthy control group. Pitch variability measures contribute to the classification but with lower relative importance compared with the most dominant MFCC features. The majority of lower-ranked features show limited influence, as reflected by SHAP values close to zero.

In the PD subgroup (TRUE = PD, *n* = 80), the feature ranking shifts again, with MFCC9_mean and MFCC2_mean emerging as the most influential predictors. Age and fundamental frequency measures also contribute prominently, followed by additional MFCC descriptors and higher-order formant-related features. Compared with the other groups, the PD subgroup exhibits a stronger concentration of importance among a smaller set of spectral features, while the remaining variables show minimal contributions.

**Fig. 6:**
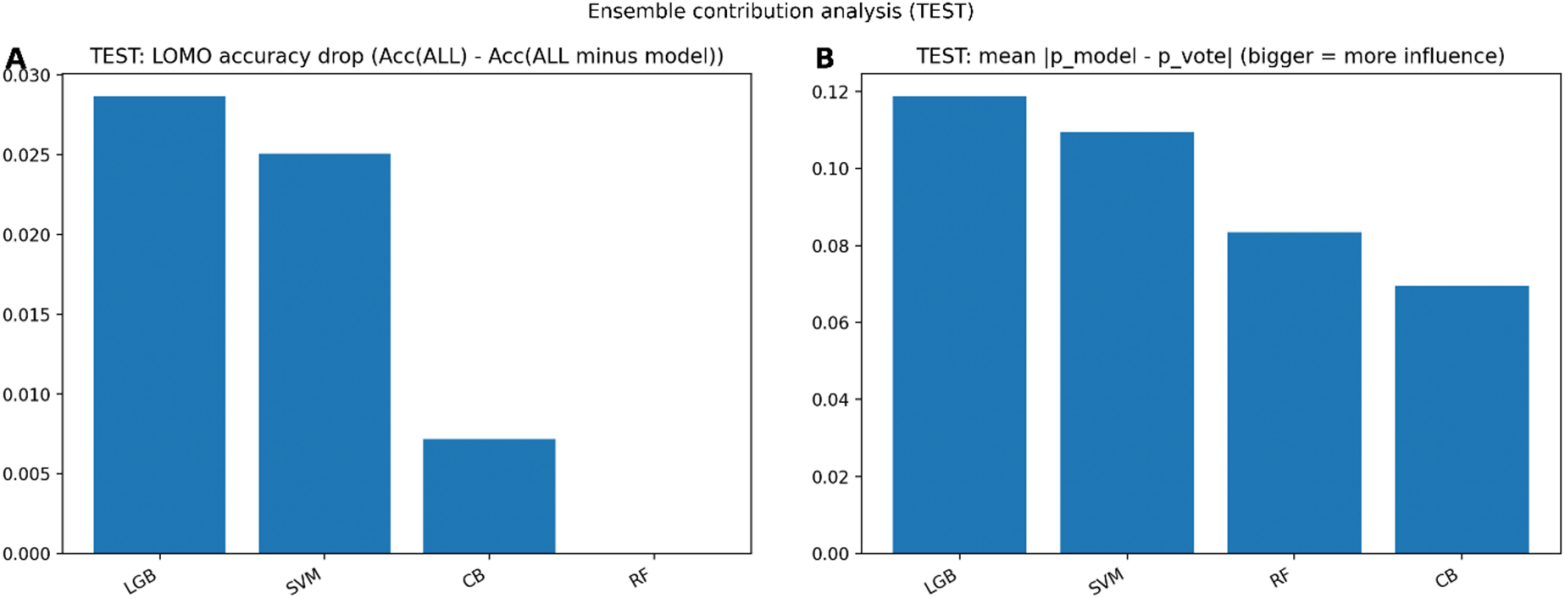
Ensemble contribution analysis for the independent test set. Panel A shows the LOMO accuracy drop, defined as the decrease in ensemble accuracy when a single base model is removed from the soft-voting classifier. Panel B shows the mean absolute deviation between each base model’s predicted class probabilities and the ensemble-averaged probabilities, with larger values indicating greater influence on the ensemble output. Results are shown for the LGB, SVM, CB, and RF classifiers.

Figure 6 summarizes the contribution of each base learner to the soft-voting classifier on the independent test set using two complementary analyses. Panel A presents the leave-one-model-out (LOMO) accuracy drop, quantified as the difference between the ensemble accuracy using all models and the accuracy obtained when one model is removed. The largest reduction in accuracy is observed when LGB is excluded, followed by the SVM, indicating that these two models contribute most strongly to the ensemble’s test performance. Removal of CatBoost results in a smaller decrease in accuracy, while removal of the RF produces a negligible change in accuracy, suggesting a limited unique contribution from this model in the ensemble. Panel B shows the mean absolute deviation between each base model’s predicted class probabilities and the ensemble’s average probabilities. Consistent with the LOMO results, LGB exhibits the highest mean absolute deviation, followed by the Support Vector Machine, indicating that these models exert the strongest influence on the ensemble’s probabilistic output. RF and CB display lower deviations, reflecting a closer alignment with the ensemble prediction and a smaller individual impact. Together, the two panels demonstrate that LGB and the SVM are the primary drivers of the ensemble’s predictive behavior on the test set, whereas CB and particularly RF contribute less independently to the final ensemble decision.

## Discussion

This study evaluated whether a soft-voting machine learning framework applied to sustained vowel “a” phonation can discriminate between PD, COPD, and HC across linguistically and clinically distinct cohorts. Motivated by the absence of widely adopted objective biomarkers for PD and COPD and by the growing interest in voice as a non-invasive digital biomarker, the work aimed to assess whether shared and condition-specific acoustic characteristics can be captured within a unified multiclass classification framework. The main findings of the present study can be summarized as follows:

1. The proposed soft-voting machine learning framework, integrating SVM, RF, CB, and LGB, achieved robust multiclass discrimination between PD, COPD, and HC on a participant-disjoint independent test set, with stable performance across training, validation, and test subsets.
2. Classification performance differed across diagnostic groups, with PD showing the highest performance, HC intermediate performance, and COPD comparatively lower performance. Misclassifications were primarily observed between HC and COPD, whereas confusion between PD and COPD was minimal.
3. Feature importance analysis using SHAP demonstrated class-dependent relevance patterns, with different subsets of acoustic features contributing to model predictions for PD, COPD, and HC rather than a single dominant feature set.
4. Vowel-space analysis based on F1–F2 distributions revealed subtle but consistent group-level differences in centroid location and dispersion, supporting physiologically grounded distinctions in phonatory and articulatory behavior across groups.

### Interpretation of classification performance

The results demonstrate that the proposed soft-voting machine learning framework, combining SVM, RF, CB, and LGB, achieved robust multiclass discrimination on a participant-disjoint independent test set. Performance differences across classes were observed, with PD showing higher classification performance than COPD, while HC exhibited intermediate behavior. These findings are consistent with the notion that neurological voice impairments may produce more stable and distinctive acoustic signatures than respiratory-driven alterations, which are known to exhibit greater heterogeneity across individuals and disease stages^35,62–64^. The comparatively lower performance for COPD may be explained by the multifactorial nature of respiratory-driven voice alterations. In COPD, phonatory characteristics are influenced not only by airflow limitation but also by compensatory vocal strategies, disease severity, comorbidities, and variability in respiratory support across recordings^26,65,66^. Such heterogeneity may reduce the consistency of acoustic patterns captured by sustained vowel “a” phonation, thereby increasing overlap with HC. In contrast, PD-related voice impairments are primarily driven by central motor dysfunction affecting phonatory control, which may result in more consistent acoustic deviations across individuals and recordings. This interpretation is supported by the confusion matrix analysis, which showed that misclassifications were predominantly observed between HC and COPD, whereas confusion between PD and COPD was minimal. The limited overlap between PD and COPD predictions suggests that the model captured disorder-specific acoustic patterns despite the presence of shared phonatory impairments, indicating sensitivity to differences in underlying physiological mechanisms.

### Cross-linguistic and cross-condition considerations

By integrating Swedish-speaking COPD data and English-speaking PD data recorded under comparable mobile conditions, the study extends prior voice-based classification research beyond traditional binary comparisons. Previous studies have predominantly focused on distinguishing a single disorder from HC, most commonly PD^23,34,67^, while respiratory-driven voice disorders have rarely been examined within the same analytical context^21^. The present approach, therefore, addresses an important gap by explicitly examining neurological and respiratory mechanisms that affect phonation within a single, explainable machine learning model. The relevance of this comparison lies not primarily in a routine direct differential diagnosis between COPD and PD, but in testing whether a voice-based framework can distinguish between different pathophysiological sources of vocal impairment rather than merely detecting non-specific abnormal phonation. Because the present study also combines cohorts from different language backgrounds, it further provides an initial indication of whether more generalizable voice-based models may be achievable across disorders, languages, and recording contexts. Additionally, a multilingual approach enables the evaluation of whether disease-related acoustic patterns generalize across linguistic contexts, rather than reflecting language-specific phonetic structures. Sustained vowel “a” phonation provides a linguistically minimal speech task, reducing lexical and prosodic variability and allowing comparison of phonatory and articulatory mechanisms across languages^61,68,69^. By combining Swedish-speaking and English-speaking cohorts, the present work examines whether acoustic alterations associated with PD and COPD persist despite differences in language background, thereby strengthening the robustness of voice-based digital biomarkers derived from mobile recordings, which previous studies have shown to contain sufficient information for effective diagnostic classification^26,70,71^.

This multi-linguistic design is particularly relevant for voice-based machine learning studies, as many reported models are trained and evaluated within a single language and may implicitly capture language-dependent characteristics^72^. In the present study, multiclass discrimination was achieved across datasets collected in different languages and representing distinct disorder groups. While the absence of misclassification between PD and COPD may partly reflect differences in language background in addition to underlying disease mechanisms, the results nevertheless indicate that a soft-voting machine learning framework can be trained and adapted to heterogeneous linguistic and clinical contexts. This consideration is also relevant for the vowel-space analysis, where the subtle centroid shifts and overlap patterns may reflect a combination of disease-related vocal differences and language-specific articulatory structure. Additionally, these findings suggest that voice-based models incorporating physiologically grounded acoustic features can retain discriminative capacity across languages and disorder types, supporting their potential applicability across populations and recording settings, while also highlighting the need for future studies explicitly designed to disentangle language and disease effects.

### Feature relevance and physiological interpretation

SHAP-based feature attribution analysis revealed that different subsets of acoustic features contributed to classification across diagnostic groups. Age, MFCC-derived spectral descriptors, and pitch-related measures consistently appeared among influential features, but their relative importance and contribution directions differed across PD, COPD, and HC. This class-dependent relevance pattern indicates that the model did not rely on a single dominant feature or feature group but instead integrated multiple acoustic cues in a group-specific manner. This use of SHAP for model interpretability follows established methodology for additive feature attributions in machine learning models^73^.

From a physiological perspective, the prominence of spectral/cepstral features (MFCCs) and formant-related measures in the COPD subgroup is consistent with evidence that COPD is associated with altered voice characteristics and increased heterogeneity, including changes in voice quality and spectral structure in sustained-vowel “a” recordings^35,74^. In contrast, the higher relative contribution of pitch-related measures and specific MFCC components in the PD subgroup is consistent with current reviews describing PD-related impairment of speech motor control and the resulting acoustic alterations observed in voice recordings^36,61,62^. Importantly, SHAP-based relevance should be interpreted as reflecting model-driven feature contributions rather than statistical significance. These relevance patterns can be considered alongside results from conventional statistical analyses as complementary perspectives. Statistical testing identifies acoustic features that differ systematically between groups at the population level, whereas SHAP highlights which features the trained model leverages most strongly to achieve multiclass discrimination in a multivariate setting.

The vowel-space analysis further complements this interpretation. Although substantial overlap was observed across groups, consistent shifts in centroid location and differences in dispersion were present, particularly for COPD relative to HC. These subtle but systematic differences align with known alterations in articulatory configuration and vocal tract control associated with respiratory and neurological disorders^12,34,36,62,75^, reinforcing the physiological plausibility of the model’s predictions.

### Methodological considerations, limitations, and strengths

Several methodological considerations should be taken into account when interpreting the findings of this study, and some of these aspects also represent strengths in a proof-of-concept setting.

First, the independent test subset was participant disjoint, ensuring that the final generalization assessment was not affected by subject leakage. Within the development cohort, the recording level split between training and validation implies that some participants contributed recordings to both subsets. This can inflate validation estimates compared with a fully participant-disjoint validation design. However, using multiple recordings per participant in model development reflects typical mobile health recording behavior and supports learning of stable acoustic patterns from repeated phonation measures, which is relevant for voice-based biomarker research using mobile recordings^41,71^.

Second, the number of unique participants in the matched cohorts was modest despite a comparatively large number of recordings. This limits coverage of inter-individual variability and constrains generalizability. At the same time, the relatively high number of repeated recordings increases the stability of feature estimation within subjects and supports proof-of-concept evaluation before larger clinical validation. Similar study designs are common in voice-based machine learning literature, and the need for larger and more standardized datasets remains a recurring conclusion across reviews in this field^21,24,28,34,63,74^.

Third, the cross-sectional design prevents inference regarding progression or longitudinal stability of the observed acoustic patterns. However, cross-sectional evaluation is appropriate for establishing baseline multiclass discrimination and for testing whether disease groups can be separated under controlled feature extraction and evaluation procedures. This is also consistent with broader methodological recommendations emphasizing unbiased evaluation frameworks, such as nested cross-validation for model selection and performance estimation when tuning is required^45,46^.

Fourth, the multi-linguistic setup is both a limitation and a strength. It does not allow complete separation of language effects from disorder effects, which should be considered when interpreting the very low confusion between PD and COPD. Nevertheless, the use of sustained vowel “a” phonation reduces lexical and prosodic variability and supports physiologically grounded comparisons across cohorts^61^. In addition, mobile voice-based studies commonly report variability across devices and acquisition contexts, and demonstrating robust performance across heterogeneous recording sources is relevant for real-world deployment^71,72^.

Fifth, SHAP-based feature attribution supported interpretation of model behavior by identifying which acoustic variables the trained model relied upon across groups^56^. This improves transparency and supports model auditing; however, these relevance patterns should be interpreted strictly as *model-based contributions* within the trained classifier rather than as causal or diagnostic evidence. Importantly, uncertainty related to clinical ground truth further constrains interpretation. The present study had no direct control over how Parkinson’s disease diagnoses were established, nor over disease stage at the time of recording. Diagnostic accuracy for Parkinson’s disease in clinical practice has been reported to be approximately 80%, with the highest uncertainty occurring in early disease stages and common misclassification against atypical parkinsonism and essential tremor^76^. In addition, population-based evidence suggests that a non-negligible proportion of individuals without a clinical diagnosis may harbor prodromal α-synuclein pathology, potentially affecting control groups (≈ 8% prevalence)^77^.

Consequently, observed SHAP patterns may partially reflect latent or misclassified pathology rather than purely group-defining physiological differences. For physiological interpretation, feature relevance should therefore be read in conjunction with established knowledge on disorder-related acoustic changes in Parkinson’s disease and chronic obstructive pulmonary disease, as well as with the broader voice biomarker literature^9,12–14,24,34,35,74,75^. From this perspective, explainability analyses are best viewed as hypothesis-generating tools that can guide future clinically controlled studies rather than as standalone evidence of disease-specific mechanisms.

### Implications and future directions

The results of the present study highlight the feasibility of applying a soft voting machine learning framework to sustained vowel “a” phonation for multiclass discrimination between PD, COPD, and HC using mobile voice recordings. The integration of interpretable acoustic features and explainability analysis demonstrates that voice-based models can capture both shared and condition-specific characteristics across neurologically and respiratory-driven voice impairments within a unified analytical framework.

Notably, diagnostic uncertainty is greatest in the earliest stages of Parkinson’s disease, when clinical symptoms are mild and conventional diagnostic approaches are least reliable. If voice-based analysis proves sensitive to subtle phonatory and articulatory alterations in this prodromal or early-stage population, such methods could provide substantial clinical value as complementary screening or monitoring tools. The present findings therefore motivate further investigation of voice analysis specifically in early and preclinical disease stages, where non-invasive, low-burden digital biomarkers are most urgently needed.

For COPD specifically, these findings support the potential of voice analysis as a complementary non-invasive digital biomarker for respiratory-related vocal impairment. Beyond diagnostic group discrimination, future work may explore whether voice-derived features are sensitive to disease severity, symptom burden, treatment-related change, or longitudinal fluctuations in respiratory function. Such directions may be particularly relevant in mobile or remote monitoring settings, where repeated low-burden voice sampling could complement established clinical measures.

The multi-linguistic and cross-condition design illustrates that voice-based classification models can be evaluated across heterogeneous recording contexts when linguistically minimal speech tasks are employed. This supports the possibility that future voice-based models may become more generalizable across disorders and languages, provided that they are validated in larger and more diverse datasets. This supports further exploration of multiclass voice-based frameworks in settings that more closely reflect real-world data acquisition, including mobile and remote monitoring scenarios. At the same time, the observed performance patterns underscore the importance of explicitly accounting for linguistic background, clinical heterogeneity, and recording variability when designing and interpreting such models.

Future investigations may extend this framework by incorporating larger and more diverse participant cohorts, additional speech tasks, and longitudinal recordings to evaluate temporal stability and progression-related acoustic changes. For COPD in particular, this should include severity-stratified cohorts and comparison with established respiratory indicators, with the aim of determining whether voice features may be informative not only for classification but also for symptom monitoring and functional disease assessment. Of particular interest is the inclusion of individuals undergoing clinical evaluation for suspected neurological or respiratory disease but who have not yet received a definitive diagnosis. Studying such diagnostically uncertain cohorts would enable assessment of whether voice-based models can support early-stage differentiation, assist clinical decision-making, or contribute to improved diagnostic accuracy in challenging cases.

Given the relatively high sensitivity observed for Parkinson’s disease in the present framework, future work may also explore the potential of voice analysis as a complementary rule-out tool, where a low predicted probability could help identify individuals unlikely to have disease and thereby reduce unnecessary follow-up investigations. Such applications would require careful calibration, prospective validation, and close integration with established clinical workflows.

Methodological extensions may further include a systematic comparison of participant-disjoint validation strategies and task-specific feature representations to characterize generalization behavior under realistic clinical conditions. In addition, combining voice-derived acoustic features with other digital or biological biomarkers, such as motor assessments, wearable sensor data, imaging, or fluid-based markers, represents a promising direction for improving robustness and diagnostic utility. Continued integration of statistical analysis and model-based interpretability may support a deeper understanding of how physiologically grounded acoustic features contribute to classification across diagnostic groups and multimodal contexts.

## Conclusion

This study demonstrates that a soft voting machine learning framework applied to sustained vowel phonation can discriminate between PD, COPD, and HC across linguistically and clinically distinct cohorts. By integrating BLA features and MFCC-based spectral features with an ensemble of complementary classifiers, the proposed approach achieved robust multiclass performance on a participant-disjoint independent test set.

The results show that classification performance differs across diagnostic groups, with Parkinson’s disease exhibiting more stable and distinctive acoustic patterns than chronic obstructive pulmonary disease, and misclassifications occurring primarily between healthy controls and chronic obstructive pulmonary disease. Feature attribution analysis revealed class-dependent relevance patterns, indicating that the model relied on different subsets of acoustic features across groups rather than a single dominant feature set. Complementary vowel space analysis further supported the presence of subtle but consistent group-level differences in articulatory and phonatory behavior.

In conclusion, the findings support the feasibility of voice-based multiclass classification frameworks that integrate interpretable acoustic features, ensemble learning, and explainability. The study contributes evidence that sustained vowel recordings collected under mobile conditions contain physiologically meaningful information capable of distinguishing between neurologically and respiratory-driven voice impairments within a unified analytical framework.

## Data Availability

The mPower dataset analyzed during the current study is publicly available through the Synapse platform maintained by Sage Bionetworks, subject to data use agreements. Link to mPower data set: https://www.synapse.org/Synapse: syn4993293/wiki/247859 . The raw voice recordings from the COPDVD dataset cannot be made publicly available due to ethical restrictions and applicable data protection regulations, including the General Data Protection Regulation. However, an anonymized version of the extracted acoustic feature dataset used in the present study can be made available from the corresponding author’s institution upon reasonable request and subject to ethical approval. The machine learning framework used in this study, including model training script, is publicly available on GitHub at https://github.com/AlperIDR/Code/blob/main/voting_Multi_Classs_GPU_04.py#L231.

## Appendix

See Table 3.

**Table 3:** The 102 acoustic and demographic features used in the experiment.

Feature group	Variables	Definition
Demographic features	Age; Gender	Participant age in years and self-reported biological sex (encoded as a binary categorical variable).
Fundamental frequency	F0_mean; F0_std	Mean and standard deviation of the fundamental frequency, reflecting average pitch level and pitch variability during sustained phonation.
Harmonics-to-noise ratio	HNR	Ratio between periodic (harmonic) energy and noise components of the voice signal, reflecting voice quality and breathiness.
Jitter measures	Local_Jitter; Local_absolute_Jitter; Rap_Jitter; PPQ_Jitter; DDP_Jitter	Cycle-to-cycle variations in fundamental frequency, quantifying short-term instability of vocal fold vibration.
Shimmer measures	Local_Shimmer; Local_db_Shimmer; APQ3_Shimmer; APQ5_Shimmer; APQ11_Shimmer; DDA_Shimmer	Cycle-to-cycle variations in amplitude of the voice signal, reflecting instability in vocal fold vibration amplitude.
Formant frequencies	F1_mean–F4_mean	Mean frequencies of the first four formants, representing resonant characteristics of the vocal tract and articulatory configuration.
Formant frequencies	F1_median–F4_median	Median frequencies of the first four formants, providing robust central estimates of vocal tract resonances.
MFCCs	MFCC1_mean–MFCC13_mean	Mean Mel-Frequency Cepstral Coefficients capturing the average spectral envelope of the voice signal.
Delta MFCCs	DMFCC1_mean–DMFCC13_mean	Mean first-order temporal derivatives of MFCCs, reflecting dynamic changes in the spectral envelope.
Delta–delta MFCCs	D2MFCC1_mean–D2MFCC13_mean	Mean second-order temporal derivatives of MFCCs, capturing acceleration of spectral changes over time.
MFCCs	MFCC1_std–MFCC13_std	Standard deviation of MFCCs, representing variability of the spectral envelope during phonation.
Delta MFCCs	DMFCC1_std–DMFCC13_std	Standard deviation of first-order MFCC derivatives, reflecting variability in spectral dynamics.
Delta–delta MFCCs	D2MFCC1_std–D2MFCC13_std	Standard deviation of second-order MFCC derivatives, reflecting variability in spectral acceleration.

## Abbreviations

PD: Parkinson’s disease
COPD: Chronic obstructive pulmonary disease
HC: Healthy controls
ML: Machine learning
SVM: Support Vector Machine
RF: Random Forest
CB: CatBoost
LGB: Light Gradient Boosting
MFCC: Mel Frequency Cepstral Coefficients
BLA: Baseline acoustic features
ROC: Receiver operating characteristic
AUC: Area under the curve
PR: Precision recall
SHAP: Shapley Additive Explanations
LOMO: Leave one model out
